# Immune checkpoint inhibitors in resectable and locoregionally advanced HNSCC: past lessons, present evidence, and future directions

**DOI:** 10.1093/oncolo/oyag366

**Published:** 2026-09-17

**Authors:** Kaewta Jeerapradit, Bhumsuk Keam, Nuttapong Ngamphaiboon

**Affiliations:** Division of Medical Oncology, Department of Medicine, Faculty of Medicine Ramathibodi Hospital, Mahidol University, 270 Rama VI Road, Ratchathewi, Bangkok, 10400, Thailand; Chakri Naruebodindra Medical Institute, Faculty of Medicine Ramathibodi Hospital, Mahidol University, 111 Moo 14, Bang Pla, Bang Phli, 10540, Thailand; Department of Internal Medicine, Seoul National University Hospital, Seoul, 03080, Republic of Korea; Division of Medical Oncology, Department of Medicine, Faculty of Medicine Ramathibodi Hospital, Mahidol University, 270 Rama VI Road, Ratchathewi, Bangkok, 10400, Thailand

**Keywords:** immune checkpoint inhibitor, head and neck squamous cell carcinoma, HNSCC, locally advanced, locoregionally advanced, NIVOPOSTOP, KEYNOTE-689, pembrolizumab, nivolumab, postoperative

## Abstract

The integration of ICIs has reshaped therapeutic strategies in head and neck squamous cell carcinoma (HNSCC). ICIs are established in recurrent/metastatic (R/M) HNSCC and have been extensively evaluated in resectable and locoregionally advanced (LA) disease, without success until the recent KEYNOTE-689 and NIVOPOSTOP studies. These pivotal trials demonstrate that perioperative and adjuvant ICIs can improve clinical outcomes and change clinical practice. Their implementation in resectable HNSCC may raise several unanswered questions, including optimal sequencing with surgery and radiotherapy, patient selection, and management of patients who progress to R/M disease after (neo)adjuvant therapy. This review summarizes the most recent evidence on ICIs in LA-HNSCC, highlighting both successes and challenges in clinical development, clinical practice implications, unresolved questions, and emerging strategies. We provide an overview of ongoing studies exploring ICI-based approaches and combinations for neoadjuvant adaptive response, surgical strategies including surgical conversion and organ preservation, and radiotherapy optimization. Additionally, we review ongoing clinical trials evaluating ICIs in combination with chemotherapy, antibody—drug conjugates, targeted therapy, radiotherapy—including stereotactic body radiotherapy (SBRT), novel ICIs, and therapeutic cancer vaccines targeting HPV or tumor neoantigens.

Implications for practice Recent phase 3 studies, including KEYNOTE-689 and NIVOPOSTOP, show that immunotherapy can improve outcomes in selected patients with resectable HNSCC, reshaping current treatment approaches. Choosing between these two approaches in clinical practice remains an ongoing debate. This review outlines the current clinical implications, key limitations, and unanswered questions in practice, including optimal patient selection and treatment selection. We also review prior unsuccessful ICI studies in locally and locoregionally advanced disease and highlight ongoing phases II-III trials of novel combinations, personalized strategies, and blood-based biomarkers to better identify high-risk patients and guide future treatment directions.

## Background

Head and neck squamous cell carcinoma (HNSCC) accounts for approximately 90% of head and neck cancers.^1^ Approximately 50%-60% of patients present with locoregionally advanced (LA) disease.^2^^,^^3^ For resectable HNSCC, standard management includes surgery followed by postoperative radiotherapy (PORT) with or without cisplatin.^4–7^ Definitive CRT remains the standard treatment for unresectable disease and organ-preservation approaches. Concurrent chemotherapy with cisplatin improves overall survival (OS) when added to RT and remains the standard radiosensitizing agent.^8^^,^^9^ Alternative radiosensitizing regimens are available for cisplatin-ineligible patients.^9–12^ Induction chemotherapy may be used in selected cases, but no randomized trial has proven its superiority over definitive cisplatin-CRT.^9^ Integrating ICIs and SMAC mimetics with definitive RT have not shown survival benefits.^13^ Despite advances in RT and surgery, outcomes for local and LA-HNSCC remain poor.^14^

Immunotherapy has become an important treatment option for R/M HNSCC. KEYNOTE-048 established pembrolizumab as a first-line standard of care, either as monotherapy for tumors with a PD-L1 combined positive score (CPS) ≥ 1 or in combination with chemotherapy.^15^ Pembrolizumab and nivolumab are also approved for second-line treatment in patients with R/M disease who progress after platinum-based chemotherapy.^9^^,^^12^ Incorporating ICIs into treatment for LA HNSCC may shift the therapeutic paradigm and enhance long-term outcomes. Two recent phase III trials, KEYNOTE-689 and NIVOPOSTOP, demonstrated improvements in event-free survival (EFS) and disease-free survival (DFS), respectively, with perioperative or postoperative ICIs in resectable HNSCC.^16^^,^^17^ This review summarizes and discusses recent evidence on integrating ICIs into definitive surgery and definitive CRT, as well as selected ongoing phase II and III trials. Nasopharyngeal and paranasal sinus carcinomas are not covered by this review due to their distinct biology and treatment approaches.

## Role of immunotherapy in HNSCC treated with definitive surgery

For nearly two decades, no phase III trial demonstrated a positive result in LA-HNSCC.^13^ Recently, two phase III studies evaluated ICIs in the perioperative and postoperative settings (Table 1 and Supplementary Table S1). KEYNOTE-689 assessed perioperative pembrolizumab in resectable HNSCC.^16^ Two cycles of neoadjuvant pembrolizumab followed by adjuvant pembrolizumab, in addition to standard of care (SOC), significantly improved EFS, leading to US FDA and EMA approvals for patients with PD-L1 CPS ≥ 1.^32^^,^^33^ In addition, efficacy and safety outcomes in the Asian subgroup were generally consistent with those observed in the overall study population.^34^ NIVOPOSTOP evaluated adjuvant nivolumab combined with cisplatin-RT in resected high-risk LA-HNSCC. This trial met its primary endpoint, showing improved DFS.^17^ Together, these findings represent important advances in the management of resectable LA-HNSCC and provide a framework for examining the distinct roles of preoperative and postoperative ICI strategies.

**Table 1.** Clinical trials evaluating ICIs +/- combinations in the curative setting for definitive surgery in HNSCC.

Clinical trials/Phase (*n*)	Population (Exp vs Control)	Drugs	Induction	Duration of induction	Definitive treatment	Adjuvant IO (vs control)	Total duration of IO	1EP	Outcomes (1EP/2EP)	Result interpretation (1EP)
**Perioperative setting**										
**KN689** **Phase 3** **(*n* = 714)^16^**	Resectable with cStage III-IVA OC 60% vs 61% OP 10% vs 11% HPV+ 3% vs 4% CPS ≥ 1 = 96%	Pembrolizumab	Pembrolizumab	2 cycles (6 wks)	Surgery + PORT ± cisplatin	Pembrolizumab (with concurrent)	17 cycles (1 yr)	EFS	CPS ≥ 10, ≥1, ITT EFS 0.66, 0.70, 0.73 (*P* .004, *P* .003, *P* .008) pCR^a^ 4%, 3%, 3% MPR^b^ 14%, 10%, 9% OS CPS ≥ 10: HR 0.72; p 0.04 (not significant at IA1)	Positive, US FDA approved
			Placebo			Placebo				
**CAMORAL** **Phase 2** **ChiCTR2000037980** **(*n* = 125)^18^**	Resectable LA cStage III-IVB OC 79% vs 86% OP 19% vs 15% HPV+ 5% vs 3% CPS ≥ 1 = 85%	Camrelizumab (C)	C + carboplatin + nab-paclitaxel	2 cycles (6 wks)	Surgery + PORT ± cisplatin	Camrelizumab	15 cycles	EFS	2-yr EFS 78% vs 46% EFS (ITT) HR 0.3; *P* .002 2-yr OS 92% vs 69% HR OS 0.25; *P* .0035 (NS) pCR 25% MPR 48%	Positive, high pCR/MPR
			None			–				
**Ferry** **NCT06208826** **Phase 2** **(*n* = 57)^19^**	Resected with MPR^b^/pCR^a^ after neoadjuvant chemoimmunotherapy	Toripalimab	Immunotherapy + chemotherapy	NR	Surgery	Toripalimab	15 cycles (45 wks)	DFS	1-yr DFS 92% vs 96%; p NR 1-yr OS 89% vs 100%; p NR	Negative, long-term follow-up needed
						PORT/CRT				
**INSIGHT** **NCT04995120** **Phase 2** **(*n* = 27)^20^**	Resectable laryngeal/HP	Toripalimab	Toripalimab + cisplatin + nab-paclitaxel	3 cycles (9 wks)	If SD/PD → Laryngeal preservation Surgery ± PORT/CRT If PR/CR → definitive CRT	Toripalimab	11 cycles (35 wks)	LPR	3-mo LPR 85.2% 1-yr LPR 79% 1-yr PFS 80% 1-yr OS 83%	Positive, satisfactory short-term LPR
**NIRT 2-HNC** **NCT04938609** **Phase 2** **(*n* = 22)^21^**	p16-negative resectable cStage III-IVA	Pembrolizumab lymphatic-sparing SBRT (GTV)	SBRT + Pembrolizumab	3 cycles (9 wks)	Surgery followed by risk-adapted PORT/CRT	Pembrolizumab	17 cycles (1 yr)	MPR^b^	MPR^b^ 73%; *P* < .001 pRR^c^ 73%; *P* < .001 Surgical de-escalation 50% PORT was omitted 77%	Positive, high MPR^b^
**NCT06444009** **Phase 2** **(*n* = 13)^22^**	Resectable OC 54% OP 15% HPV+ 7.5% Larynx 30%	Ivonescimab (I) (Anti PD-1/VEGF) Cadonilimab (C) (Anti PD-1/CTLA4) Penpulimab (P)	Cohort 1 I + cisplatin + nab-paclitaxel (Chemo)	3 cycles (9 wks)	Surgery + PORT ± cisplatin ^d^ If pCR^a^ → PORT can be omitted	Ivonescimab	16 cycles (48 wks)	pCR^a^	pCR^a^ Cohort 1/2/3: 80%/0%/67% MPR^b^ Cohort 1/2/3: 80%/100%/67%	Positive, high pCR^a^/MPR^b^(small sample size)
			Cohort 2 C + Chemo			Cadonilimab				
			Cohort 3 P + Chemo			Penpulimab				
**Neoadjuvant setting (Induction)**										
**NCT05522985** **Phase 2** **(*n* = 122)^23^**	Resectable stage III or IV	Toripalimab (T)	T + Chemo	3 cycles (9 wks)	Surgery + PORT/CRT	-	3 cycles (9 wks)	pCR^a^	pCR^a^ 58% vs 35%; *P* .030 MPR^b^ 82% vs 53%; *P* .004 2-yr DFS 87% vs 72% 2-yr OS 91% vs 78%	Positive, high pCR^a^/MPR^b^
			Cisplatin + nab-paclitaxel (Chemo)							
**NeoCPC** **NCT04826679** **Phase 2** **(*n* = 48)^24^**	Resectable OP 29% CPS ≥ 1: 79%	Camrelizumab (C)	C + cisplatin + nab-paclitaxel	3 cycles (9 wks)	Surgery + PORT/CRT	–	3 cycles (9 wks)	ORR^e^	pCR^a^ 56% MPR^b^ 63% ORR^e^ 90%	Positive, high ORR^e^/pCR^a^
**ChiCTR2200060094** **Phase 2** **(*n* = 45)^25^**	LA hypopharyngeal SCC CPS ≥ 1: 89%	Tislelizumab (T)	T + cisplatin + nab-paclitaxel	2 cycles (6 wks)	If SD/PD → Laryngeal preservation Surgery ± PORT/CRT If PR/CR → definitive CRT	–	2 cycles (6 wks)	3-mo LPR	3-mo LPR 87% pCR^a^ 21% ORR^e^ 62% 1-yr PFS 70% 1-yr OS 82%	Positive, favorable short-term LPR.
**NeoRTPC02** **NCT05343325** **Phase 2** **(*n* = 28)^26^**	Resectable stage III-IVB OP 14% HPV+ 46% CPS ≥ 1 57%	Tislelizumab (T) Low-dose RT (LDR, 8 Gy/8F, 4F/cycle)	T + cisplatin + nab-paclitaxel + LDR	2 cycles (6 wks)	Standard of care surgery	–	–	pCR^a^	pCR^a^ 61% MPR^b^ 22% ORR^e^ 64%	Positive, high pCR^a^
**NeoCHANCE-1** **NCT05517330** **Phase 2** **(*n* = 25)^26^**	Resectable OP 28% HPV+ 28% CPS ≥ 1: 72%	Tislelizumab Afatinib	Tislelizumab + afatinib	2 cycles (6 wks)	Standard of care surgery	–	2 cycles (6 wks)	MPR^b^	pCR^a^ 17% MPR^b^ 35% ORR^e^ 48% Grade 3-4 adverse events: diarrhea (20%), hypokalemia (16%), rash (12%)	Positive, high MPR^b^
**NCT03342911** **Phase 2** **(*n* = 27)^27^**	Resectable OC 73%, OP 73% HPV+ 15%	Nivolumab (N)	N + carboplatin + paclitaxel	3 cycles (6 wks)	Standard of care surgery	–	–	pRR^c^	pCR^a^ 42% pRR^c^ 69%	Positive, high pRR^c^
**Adjuvant setting in definitive surgery/multimodal treatments**										
**NIVOPOSTOP** **Phase 3** **(*n* = 666)^17^**	Resected pStage III-IVB with high-risk features OC 58% vs 58% OP 17% vs 18% HPV+ 5% vs 5% CPS ≥ 1: 81% ECE/R1 57% vs 59%	Nivolumab (N)	None		Surgery + PORT + cisplatin + N	Nivolumab	9 cycles (9 mos)^d^	DFS (ITT)	DFS HR 0.76; *P* .034	Positive
					Surgery + PORT + cisplatin	Placebo				
**IMvoke010** **Phase 3** **(*n* = 406)^28^**	Stage IVA-IVB s/p definitive treatment OP 35% vs 38% HPV+ 17% vs 17% TAP ≥ 5%: 78%	Atezolizumab			Surgery (38%) Definitive RT/CRT (62%)	Atezolizumab	16 cycles (1 yr)	EFS	EFS HR 0.94; *P* .68 Median EFS 59.5 vs 52.7 mo. OS HR 0.96 (0.68-1.36) 2-yr OS 82% vs 79.2%	Negative
						Placebo				
**ADRISK** **NCT03480672** **Phase 2** **(*n* = 204)^29^**	Intermediate to high risk resected pStage III-IVA/B OP 66% vs 66% HPV+ 45% vs 46% CPS ≥ 1: 93% High (64%), intermediate (36%) risk	Pembrolizumab (P)			Surgery + PORT + cisplatin + P	Pembrolizumab	1 yr	EFS	EFS HR 0.81; *P* .423 OS HR 0.85; *P* .591	Negative
					Surgery + PORT + cisplatin	–				
**IMPORT** **NCT04523883** **Phase 2** **(*n* = 87)^30^**	Cisplatin-ineligible, high-risk resected pStage III-IVA, age > 65 yrs	Toripalimab			Surgery	RT-Toripalimab	3 cycles (9 wks) - concurrent	DFS	2-yr DFS 55% vs 51%; *P* .866	Negative
						PORT				
**NCT03715946** **Phase 2** **(*n* = 41)^31^**	Resected high risk p16+ OPSCC pStage III-IVA	Nivolumab (N)			Surgery + N-RT	Nivolumab	7 mos (q2w or q4w)^d^	PFS	3-yr PFS 86% 3-yr OS 86%	Positive, but target population not yet reached

a pCR = pathological complete response.

b MPR = major pathologic response.

c pRR = MPR + pCR.

d Including concurrent RT phase.

e ORR = objective response rate.

### Preoperative strategies

For this review, a neoadjuvant/induction strategy refers to immunotherapy administered before surgery without planned postoperative ICI therapy, whereas a perioperative strategy includes both preoperative and postoperative ICIs. Preoperative ICI may maximize immune priming in the presence of an intact tumor, permit pathological response assessment, and limit treatment exposure.^35^ In contrast, perioperative therapy may provide more sustained systemic immune control but requires a longer treatment duration and may increase toxicity, cost, and the possibility of overtreatment. These potential benefits and limitations must therefore be carefully balanced.^36^

The potential advantages of administering immunotherapy before surgery are supported by the underlying immunobiology of HNSCC. The high risk of recurrence following definitive treatment may partly reflect tumor immunoediting, whereby immune pressure selects tumor-cell populations expressing subdominant antigens, together with T-cell exhaustion characterized by increased PD-1 expression on CD8^+^ T cells and tumor-infiltrating lymphocytes.^37^ These mechanisms provide a rationale for preoperative immunotherapy, rather than relying exclusively on postoperative treatment. While the primary tumor and tumor-draining lymph nodes remain intact, preoperative immunotherapy may enhance antigen presentation, promote expansion of tumor-specific T-cell clones, facilitate immune priming within tumor-draining lymph nodes, broaden polyclonal T-cell responses, and activate tissue-resident memory T cells, thereby strengthening systemic immune surveillance and micrometastatic disease control.^37–41^

#### Perioperative approach

In addition to KEYNOTE-689, several phase II trials have evaluated perioperative ICIs combined with chemotherapy (Table 1). These strategies generally comprise short-course neoadjuvant chemoimmunotherapy, followed by definitive surgery and PORT with or without cisplatin, and maintenance ICIs. The phase II CAMORAL study assessed neoadjuvant camrelizumab with chemotherapy, followed by surgery, SOC, and adjuvant camrelizumab (without concurrent RT phase).^18^ This regimen improved EFS, reducing both local recurrence and distant metastasis, with a pCR rate of 25%, compared with 3%-4% in KEYNOTE-689. Other small studies combining ICIs with platinum-based chemotherapy^20^^,^^22^ or bispecific antibodies^22^ targeting PD-1/VEGF or PD-1/CTLA-4 have also reported promising pCR rate, including a potential organ-preservation approach.^20^

#### Neoadjuvant/induction-only approach

Neoadjuvant ICIs, alone or combined with chemotherapy, targeted therapy, or preoperative RT, have shown promising activity (Table 1). Several phase II studies evaluating short-course neoadjuvant chemoimmunotherapy before curative surgery reported substantial pCR rates, with one also exploring organ preservation in LA hypopharyngeal squamous cell carcinoma (HPSCC).^23–25^^,^^27^^,^^42^ Other approaches have combined ICIs with targeted therapy or low-dose preoperative RT.^26^^,^^43^ PORT was generally guided by conventional pathological risk factors per SOC, without planned continuation of ICIs after surgery, thereby distinguishing these studies from perioperative strategies. Overall, these findings remain hypothesis-generating because of heterogeneous regimens, limited sample sizes, and immature follow-up precluding definitive conclusions regarding long-term efficacy or the optimal treatment approaches.

### Postoperative/adjuvant strategy

NIVOPOSTOP demonstrated that adding nivolumab to postoperative cisplatin-CRT improved investigator-assessed DFS without a neoadjuvant component, in contrast to the perioperative strategy used in KEYNOTE-689.^16^^,^^17^ However, the DFS benefit did not reach statistical significance by blinded independent central review, warranting cautious interpretation of about NIVOPOSTOP findings. Other postoperative ICIs trials have been previously unsuccessful (Table 1). The phase III IMvoke010 study of adjuvant atezolizumab failed to improve survival outcomes.^28^ Although most patients (62%) received definitive RT/CRT, 38% treated with definitive surgery. Similarly, the phase II ADRISK study, designed akin to NIVOPOSTOP, evaluated pembrolizumab plus standard PORT with cisplatin but did not show an EFS benefit. This result may partly reflect its comparatively favorable risk population, which included patients with intermediate pathological risk and HPV-related OPSCC.^29^ Although ADRISK and KEYNOTE-689 evaluated the same ICI for a similar duration, their differing outcomes may partly reflect preoperative immune priming in KEYNOTE-689, which was absent in ADRISK. However, this hypothesis remains speculative and cannot be confirmed through indirect cross-trial comparisons.

Two additional phase II studies explored postoperative radioimmunotherapy with different goals, including RT de-escalation in resected, high-risk, p16-positive LA-OPSCC and for cisplatin-ineligible patients. However, both were small, early-phase studies, and one was underpowered, limiting definitive conclusions.^30^^,^^31^

## Role of immunotherapy in HNSCC treated with definitive chemoradiotherapy

There is a strong biological rationale for combining ICIs with RT. RT may exert immunostimulatory effects by increasing tumor antigen release and major histocompatibility complex class I expression, activating dendritic cells, and promoting the recruitment and effector function of tumor-infiltrating lymphocytes.^44^ However, RT may also promote immunosuppression through PD-L1 upregulation, expansion or recruitment of regulatory T cells and tumor-associated macrophages, and treatment-related lymphopenia. The net effect may depend on radiation dose and fractionation, treatment volume, sequencing, and concurrent systemic treatment administered.^45^^,^^46^

A summary of phases II and III trial results is shown in Table 2. To date, no study has demonstrated a survival benefit sufficient to define a new SOC. Notably, some trials have even reported inferior outcomes with ICI-based regimens. Several factors may contribute to the limited efficacy of combining ICIs with definitive RT or CRT, including biomarker-unselected PD-L1 populations, reduced T-cell priming from extensive nodal irradiation, and the potential immunosuppressive effects of high-dose concurrent cisplatin.

**Table 2.** Clinical trials evaluating ICIs +/- combinations in the curative setting for definitive CRT in HNSCC.

Clinical trials/Phase (*n*)	Population Baseline characteristics (Exp vs Control)	Drugs	Definitive treatment	Adjuvant IO	Duration of IO	1EP	Outcomes	Result interpretation (1EP)
**Cisplatin eligible**								
**KN412** **Phase 3** **(*n* = 804)^47^**	LA unresectable OP 50% vs 51% HPV+ 27% vs 26% CPS ≥ 1: 85%	Pembrolizumab (P)	P + cisplatin-RT	Pembrolizumab	17 cycles (1 yr)^a^	EFS	Median EFS 71.8 vs 49.8 mo. EFS HR 0.79; p-NR 5-yr EFS 55% vs 47% 5-yr OS 64% vs 60%	Negative, but pembrolizumab arm showed longer EFS trends in long-term follow-up
			Placebo + cisplatin-RT	Placebo				
**CompARE** **Phase 3** **NCT04116047** **(*n* = 594)** **(planned *n* = 785)^48^**	Intermediate-high risk OPSCC Intermediate risk HPV+ 85% High risk HPV- 15% PD-L1 not reported yet	Durvalumab (D)	D + cisplatin-RT(Arm 5) (Induction with D x1 cycle)	Durvalumab	7 cycles (28 wks)	OS	3-yr OS 84% vs 82% OS HR 0.97; p-NR EFS HR 1.00; *P* .881	Negative
			Cisplatin-RT (Arm 1)	–	–			
**JAVELIN HN100** **Phase 3** **(*n* = 697)^49^**	cStage III-IVB OP 45% vs 49% HPV+ 35% vs 34% PD-L1 high (≥ 25%) (SP263) 18%	Avelumab (A)	A + cisplatin-RT	Avelumab	28 cycles (14 mos)^a^	PFS	Median PFS not reached PFS HR 1.21; *P* .920	Negative, avelumab-CRT showed detrimental effect compared to CRT-cisplatin
			Cisplatin-RT	–				
**GORTEC 2017-01 REACH** **Phase 3** **(*n* = 426)^50^**	cStage III-IVB Cohort 1, cisplatin eligible OP 70% HPV+ 49%	Avelumab (A) Cetuximab	A + cetuximab-RT	Avelumab	28 cycles (14 mos)^a^	PFS	4-yr PFS 42% vs 55% PFS HR 1.40; *P* .012 OS HR 1.45; *P* .017	
			Cisplatin-RT	–				
**NRG HN005** **Phase 2/3** **(*n* = 382)^51^**	Early-stage p16+ OPSCC with smoking ≤ 10 pack yrs	Nivolumab	Cisplatin-RT (70 Gy) – arm 1	-	-	PFS	(Arm1:2:3) 2-yr PFS: 98.1% vs 88.6% vs 90.3% HR 4.34 (Arm1 vs 2) HR 4.51 (Arm1 vs 3) 2-yr OS: 99% vs 98% vs 96%	Negative, RT de-escalation in early p16^+^ OPSCC met phase 2 interim futility criteria.
			Cisplatin-RT (60 Gy) – arm 2					
			Nivolumab + RT (60 Gy) – arm 3	Nivolumab	6 cycles (12 wks)^a^			
**CheckRad-CD8** **NCT03426657** **Phase 2** **(*n* = 120)^52^**	cStage III-IVB 80 patients enrolled HPV+ OP 29%	Durvalumab (D) Tremelimumab (T)	(Induction cisplatin + docetaxel + D + T for 1 cycle) → If increased intratumoral CD8^+^ immune cell density or pCR → D + T-RT	D + T up to 4 cycles then D alone	12 cycles (1 yr)^a^	Feasibility	feasibility rate 82% pCR^b^ 51% 2-yr PFS 72% 2-yr OS 84%	Positive feasibility with promising pCR, but high toxicity observed (≥G3: 95%)
**HCC 15-132** **Phase 2** **(*n* = 80)^53^**	LA unresectable cStage III-IVB OP 77% vs 66% HPV+ 56% vs 49% Median CPS: 15	Pembrolizumab (P)	Cisplatin-RT (sequential)	Pembrolizumab	8 cycles (24 wks)	PFS	4-yr LRC 96% vs 64% HR 0.11; *P* .012 4-yr-PFS 69% vs 49% HR 0.55; *P* .132 4-yr OS 83% vs 71% HR 0.51; *P* .170	Positive, favored sequential approach
			P + Cisplatin-RT(concurrent)		8 cycles (24 wks)^a^			
**OPTIMA II** **NCT03107182** **Phase 2** **(*n* = 56)^54^**	LA p16+ OPSCC CPS ≥ 20: 42% Low risk 51% High risk 47%	Nivolumab (N) Chemotherapy	Induction with carboplatin + paclitaxel + N for 3 cycles (9 wks) → response-adaptive LRT^c^ - A: Single modality (RT 50 Gy or TOR) - B: Intermediate Cisplatin-RTor TFHX (50 Gy) - C: Regular Cisplatin-RTor TFHX (70 Gy)	Nivolumab	6 cycles (6 mos)	DRR^d^	DRR^d^ 70% pCR^b^ 67% ORR^e^ 97% 2-yr PFS 90% 2-yr OS 91%	Negative, response-adapted LRT^c^ is feasible
**DEPEND** **NCT03944915** **Phase 2** **(*n* = 36)^55^**	p16- stage IVa/b OP 19% CPS ≥ 20: 22%	Nivolumab (N) Chemotherapy	Induction with carboplatin + paclitaxel + N for 3 cycles (9 wks) → response-stratified CRT-cisplatin/TFHX deep response: 66 Gy Non-deep response: 70 Gy	Nivolumab	9 cycles (9 mos)	DRR^d^	DRR 53% (CPS ≥1: 75% vs <1: 27%; *P* .006) ORR^e^ 86% 2-yr PFS 66% 2-yr OS 73%	Positive, response-adaptive CRT is feasible
**IMplus** **NCT04440917** **Phase 2** **(*n* = 32)^56^**	Unresectable cStage III-IVb SCC or poorly or undifferentiated OP 44%	Camrelizumab (C) Apatinib (A)	Induction with C + A for 2 cycles (8 wks) → definitive CRT	–	–	ORR^e^	ORR^e^ 83%	Positive (in the first stage)
**Cisplatin ineligible**								
**GORTEC 2017-01 REACH** **Phase 3** **(*n* = 275)^50^**	cStage III-IVB Cohort 2, cisplatin ineligible OP 61% HPV+ 35%	Avelumab (A) Cetuximab	A + cetuximab-RT	Avelumab	28 cycles (14 mos)^a^	PFS	4-y PFS rate 34% vs 18% PFS 0.80 (0.60-1.06); *P* not reported DMFS subHR 0.24 (0.11-0.49) OS HR 1.05 (0.76-1.44)	Negative, avelumab + cetuximab-RT showed favorable trend in PFS and distant metastasis, not OS
			Cetuximab-RT	–				
**NRG HN004** **Phase 2/3** **(*n* = 190)^57^**	Cisplatin-ineligible OP 57% vs 48% HPV+ 47% vs 48%	Durvalumab	Durvalumab-RT	Durvalumab	7 cycles (28 wks)^a^	PFS	Median PFS 2.2 vs 2.7 yrs PFS HR 1.43, *P* .920 2-yr OS 69% vs 78%; *P* not reported	Negative, stopped early at the interim futility analysis
			Cetuximab-RT	–				
**Pembrorad** **Phase 2** **(*n* = 131)^58^**	LA unresectable, unfit for high dose cisplatin OP 59% vs 62% HPV+ 46% vs 45% CPS ≥ 1 = 67%	Pembrolizumab	Pembrolizumab-RT	–	3 cycles (9 wks) (concurrent)	LRC	15-mo LRC 60% vs 59% HR 1.05; *P* .910 PFS HR 0.85; *P* .47 OS HR 0.83; *P* .49	Negative
			Cetuximab-RT	–				
**LCCC1509** **NCT02609503** **Phase 2** **(*n* = 29)^59^**	Cisplatin-ineligible OP 69% HPV+ 48%	Pembrolizumab	Pembrolizumab-RT	Pembrolizumab	6 cycles (18 wks)^a^	PFS	Median PFS not reached 2-yr PFS 71% 2-yr OS 75%	Positive

a Including concurrent RT phase.

b pCR = pathological complete response.

c LRT = locoregional therapy.

d DRR = deep response rate (≥50% response to induction therapy based on RECIST criteria).

e ORR = objective response rate.

### Cisplatin-eligible patients

Most trials in this setting have evaluated ICIs initiated shortly before definitive CRT as a lead-in phase, continued concurrently with definitive CRT, and followed by adjuvant maintenance therapy. Several studies have evaluated this approach in LA-HNSCC, including JAVELIN HN100 (avelumab with cisplatin-CRT),^49^ GORTEC 2017-01 REACH cohort 1 (avelumab with cetuximab-RT),^50^ and NRG-HN005 (nivolumab-RT as a de-escalation strategy in early-stage p16+ OPSCC).^51^ In all three studies, ICIs were given concurrently with RT, but each showed unfavorable outcomes.

JAVELIN HN100 was terminated for futility, whereas GORTEC 2017-01 REACH cohort 1 also failed to improve outcomes compared with standard cisplatin-CRT.^49^^,^^50^ The reasons for these detrimental findings remain uncertain. Potential explanations include treatment-related lymphopenia, irradiation of tumor-draining lymph nodes, and alterations in the TME during concurrent RT, which may counteract the immunostimulatory effects of RT and impair T-cell priming and expansion.^46^^,^^49^ The potentially limited immunostimulatory activity of PD-L1 blockade in this setting may also have contributed. In contrast, the phase III KEYNOTE-412 study evaluating concurrent and adjuvant pembrolizumab with cisplatin-CRT showed a trend toward improved EFS, although the statistical threshold for significance was not met in PDL1 all comers.^47^ However, at the 74-month follow-up, greater separation of the EFS curves was observed.

The optimal sequencing of ICIs and RT in LA-HNSCC remains an area of investigation. The randomized phase II HN 15-132 trial compared sequential vs concurrent pembrolizumab with cisplatin-based CRT and demonstrated a numerically higher 1-year PFS with the sequential approach.^53^ These findings are consistent with results from studies of durvalumab in unresectable stage III non-small-cell lung cancer, in which the PACIFIC trial showed improved PFS and OS with adjuvant durvalumab following completion of CRT.^60^ In contrast, the PACIFIC-2 study, which evaluated concurrent durvalumab with CRT followed by adjuvant therapy, did not demonstrate a comparable magnitude of benefit.^61^ However, not all sequential strategies have been successful. The phase III IMvoke010 trial—which included a majority of patients treated with definitive CRT and employed a sequential ICI approach—did not demonstrate a survival benefit with adjuvant atezolizumab.^28^ This lack of benefit may be partly explained by the heterogeneous study population, which combined both definitive CRT and surgery, and the delayed initiation of immunotherapy up to 16 weeks after CRT completion. Similarly, the phase III CompARE study, which evaluated durvalumab without a concurrent phase in intermediate-risk OPSCC, failed to improve OS, potentially reflecting the favorable prognosis of p16-positive patients.^48^

A potential explanation for these mixed results relates to differences in radiation fields and immunologic effects. In LA-HNSCC, definitive RT typically involves extensive bilateral elective nodal irradiation, which may expose tumor-draining lymph nodes and circulating lymphocytes to radiation, potentially attenuating systemic immune responses.^62^^,^^63^ Collectively, these findings suggest that ICIs may have limited activity in biomarker-unselected populations and underscore the importance of optimal sequencing with RT and patient selection. These outcomes may also reflect differences in trial design, statistical thresholds, and the absence of biomarker-driven strategies. Nevertheless, these hypotheses remain exploratory. Given the negative results of integrating ICI monotherapy into definitive CRT, recent phase II studies have explored induction ICIs combined with chemotherapy or targeted therapy.^54–56^ Although these studies demonstrated encouraging antitumor activity, their single-arm or early-phase designs preclude definitive conclusions and warrant further investigation.

### Cisplatin-ineligible patients

To date, this approach has not demonstrated a significant survival benefit. Most studies used cetuximab-RT as the comparator, with varying durations of maintenance ICI after RT. The phase III GORTEC 2017-01 REACH (cohort 2) showed no OS improvement with avelumab-cetuximab-RT.^50^ The phase II/III NRG-HN004 study of durvalumab-RT was stopped early at interim analysis for futility after showing inferior PFS.^57^ A randomized phase II study comparing pembrolizumab-RT found no improvement in LRC.^58^ In contrast, a single-arm phase II study of pembrolizumab-RT with maintenance reported a promising PFS.^59^ Thus, an effective concurrent systemic treatment for cisplatin-ineligible patients remains an unmet clinical need.

## Current clinical implications, limitations, and unanswered questions

The potentially practice-changing results of KEYNOTE-689 and NIVOPOSTOP studies have influenced the management of resectable HNSCC (Supplementary Table S1). Pembrolizumab has received US FDA and EMA approvals for resectable HNSCC patients with PD-L1 CPS ≥ 1 and has been incorporated into clinical practice guidelines, including NCCN for perioperative use in resectable stage III-IVA HNSCC.^9^^,^^32^^,^^64^ In contrast, positive results from NIVOPOSTOP has not yet received regulatory approval for this indication. Despite the positive results of both trials, several considerations remain.

In clinical practice, choosing between perioperative pembrolizumab, as in KEYNOTE-689, or upfront surgery followed by adjuvant nivolumab, as in NIVOPOSTOP, is challenging because these enrolled potentially overlapping populations and addressed different clinical decision points. The positive results of NIVOPOSTOP, in which nivolumab was added to postoperative CRT without a neoadjuvant component, provide an interesting contrast to studies such as KEYNOTE-689. These findings raise important questions regarding the relative contribution of neoadjuvant versus adjuvant immunotherapy to the overall clinical benefit observed with perioperative approaches. The NIVOPOSTOP results also prompt the question of whether neoadjuvant immunotherapy is always necessary or whether some of its proposed advantages could potentially be achieved through optimized postoperative treatment strategies. Choosing between these two approaches in clinical practice remains an ongoing debate. The use of perioperative pembrolizumab raises several concerns, particularly the risk of disease progression during the neoadjuvant phase, which may render tumors unresectable or necessitate a more extensive surgical field, as well as the potential for overtreatment in low-risk patients. Subgroup analysis of KEYNOTE-689 demonstrated an EFS benefit across both high- and low-risk postoperative groups who received PORT with or without cisplatin.^65^ However, radiographic progressive disease (PD) during the neoadjuvant phase was observed in 25% of patients (92/363); 4% (15/363) discontinued treatment due to progression, while 21% (77/363) experienced PD that did not preclude surgery. In contrast, an adjuvant approach using nivolumab in postoperative high-risk patients—without a neoadjuvant priming phase and with a shorter duration of ICI exposure compared with KEYNOTE-689—may lead to differences in recurrence patterns, including variations in locoregional vs distant failure. However, neoadjuvant pembrolizumab may be preferred in patients with a lower risk of early progression, such as those with high PD-L1 expression or more indolent tumor biology. Conversely, patients with marginally resectable or rapidly progressive tumors may derive greater benefit from upfront surgery followed by adjuvant nivolumab, as in the NIVOPOSTOP paradigm, particularly when high-risk pathological features are identified. In clinical practice, treatment decisions should be individualized, taking into account disease tempo, prior clinical course, tumor profiles, and multidisciplinary team discussion to determine the most appropriate strategy for each patient. Neoadjuvant chemoimmunotherapy may represent a future approach for patients with aggressive but still resectable disease. However, current evidence is limited to phase II studies (e.g., CAMORAL), and confirmation from randomized phase III trials is needed. Notably, most ongoing preoperative studies are focusing on combination ICI-based neoadjuvant regimens, which may achieve higher response rates compared with ICI monotherapy (see section “future directions”).

Refining patient selection also remains important. In NIVOPOSTOP, approximately 10% had multiple intermediate-risk factors (e.g., multiple PNI or ≥4 positive lymph nodes). While patients with multiple intermediate-risk features had significantly worse survival, postoperative cisplatin-based CRT did not significantly improve OS or EFS, leaving uncertainty about whether adding nivolumab benefits this subgroup.^7^ The negative ADRISK study, which enrolled a lower-risk population, further emphasizes that the benefit of ICIs may depend on refined patient selection, particularly in identifying truly high-risk populations.^29^

The use of ICIs in resectable LA-HNSCC may influence strategies for R/M HNSCC. Unlike platinum-refractory disease, there is no established definition of ICI refractoriness in this setting. In melanoma, resistance to adjuvant anti-PD-1 therapy is defined using a 12-week cutoff from the last ICI dose to distinguish primary resistance or early relapse from potentially PD-1-responsive or late relapse.^66^^,^^67^ For patients with resectable HNSCC who progress after neoadjuvant or adjuvant ICIs, it remains unclear how to implement ICIs in the R/M setting. Key questions include how to define ICI-refractory disease, whether re-biopsy for PD-L1 testing are warranted, and whether melanoma-based 12-week or HNSCC platinum-refractory 6-month cutoffs can be applied.

## Future directions

### Ongoing ICI-based clinical trials in resectable and locoregionally advanced HNSCC

The role of ICI-based approaches in non-metastatic HNSCC is under active investigation. Numerous studies are evaluating combinations with chemotherapy, antibody-drug conjugates (ADC), targeted therapy, combination immunotherapies—including with SBRT—and novel agents such as bispecific antibodies across resectable and LA HNSCC. Key areas of focus include surgical strategies, such as surgical conversion and organ preservation, and radiotherapy optimization, including omission, dose, field, and technique. A summary of these ongoing ICI studies, organized by focus area, is presented below.

#### Ongoing studies of ICIs and combinations in LA-HNSCC

Two cycles of neoadjuvant pembrolizumab achieved minimal MPR and pCR, providing a rationale for combining ICIs with other modalities to improve responses.^16^ Ongoing studies in resectable LA-HNSCC (Table 3^68–81^ and Supplementary Table S2^82–98^) are exploring induction ICI-based combinations, including chemotherapy-based regimens,^68–72^^,^^74^^,^^75^^,^^82–87^^,^^96^ ADCs,^73^ targeted therapies (tyrosine kinase inhibitors,^79^^,^^88^^,^^89^ CDK4/6 inhibitors^90^), preoperative RT,^79^^,^^91^^,^^92^ and novel ICI combinations.^76^^,^^77^^,^^80^^,^^93^ ICIs are usually continued as adjuvant maintenance; however, in KEYNOTE-689, it remains unclear whether the EFS benefit derives from the neoadjuvant, adjuvant phase, or both, and a phase II study is now evaluating benefit of adjuvant ICIs after neoadjuvant ICI.^93^ Following NIVOPOSTOP, several studies are testing other ICIs in resected high-risk LA-HNSCC to assess their benefit when added to PORT with cisplatin,^81^^,^^94^ including toripalimab in a design similar to IMvoke010.^95^

**Table 3.** Phase III and selected ongoing phase II studies of induction and/or adjuvant ICIs with definitive surgery in HNSCC.

Study	Phase (*n*)	Population	Investigational treatment	Induction	Duration of induction	Definitive treatment	Adjuvant immunotherapy	Duration of maintenance adjuvant	1EP	Recruitment status
**Induction ICI with chemotherapy-based regimen**										
**REDUCTION-I** **NCT05582265^68^**	3 (*n* = 537)	Resectable, cStage III/IVA	Anti-PD-1	Tislelizumab + short chemotherapy (1 cycle)	3 cycles (9 wks)	Surgery + PORT ± cisplatin	Tislelizumab	N/A	EFS	Recruiting
				Tislelizumab + chemotherapy	3 cycles (9 wks)		Tislelizumab			
				None	**-**		Observation	**-**		
**NEOPCOSCC** **NCT05798793^69^**	3 (*n* = 309)	Resectable LA oral SCC with cStage III-IVA		Docetaxel + cisplatin	3 cycles (6 wks)	Surgery + PORT ± cisplatin	-	-	EFS	Recruiting
				Camrelizumab + docetaxel + cisplatin						
				None						
**EFFECT-neo** **NCT06102395^70^**	3 (*n* = 272)	cStage IIIA—IVB		Pembrolizumab + platinum + taxane or 5FU	2 cycles (6 wks)	If CR → PORT/CRT If PR/SD → Surgery ± PORT/CRT If PD → standard treatment	-	-	pCR^a^	Recruiting
				Platinum + taxane or 5FU						
**TopHill** **NCT06258811^71^**	3 (*n* = 134)	Resectable LA cStage III-IVA OCSCC		Tislelizumab + nab-paclitaxel + cisplatin	2 cycles (6 wks)	Surgery + PORT/CRT	Tislelizumab	17 cycles (1 yr)	EFS	Recruiting
				None	–		–	–		
**2024-SR-421** **NCT06699498^72^**	2 (*n* = 45)	Resectable cStage III/IVA	Anti-PD-L1 Multi-target kinase TKI	Benmelstobart + anlotinib + cisplatin + albumin-bound paclitaxel	3 cycles (9 wks)	Surgery + PORT/CRT	Benmelstobart if ctDNA+	N/A	MPR^b^	Not yet recruiting
**NCT06530914^73^**	2 (*n* = 30)	Resectable cStage III-IVB LA-EGFR-positive	Anti-PD-1 ADC	MRG003 (Anti-EGFR with MMAE payload) + pucotenlimab	3 cycles (9 wks)	Surgery	Pucotenlimab	8 cycles (24 wks)	pCR^a^	Not yet recruiting
**TRENT-003** **NCT06894459^74^**	2 (*n* = 180)	Resectable LA larynx/HP	Anti-PD-1 Response adaptive PORT	Pembrolizumab + cisplatin + nab-paclitaxel	3 cycles (9 wks)	Surgery: pCR^a^: PORT 50-56 Gy alone non-pCR^a^, no high-risk factors: CRT (56-60 Gy) – cisplatin non-pCR^a^ with high-risk factors: CRT (66-70 Gy) – cisplatin	–	–	pCR^a^	Not yet recruiting
**NCT06306846^75^**	2 (*n* = 81)	Resectable	Anti-PD-1 Anti-EGFR Pre-op SBRT Response adaptive PORT	anti-PD-1 + chemotherapy + SBRT	2 cycles (6 wks)	Surgery + PORT/CRT ^c^If pCR^a^/MPR^b^ → PORT can be omitted	anti-PD-1	6 cycles (12 wks)	MPR^b^	Recruiting
				anti-PD-1 + chemotherapy + cetuximab			anti-PD-1			
				anti-PD-1 + chemotherapy			anti-PD-1			
**Induction ICI and targeted therapy and ICI combination (non-chemotherapy regimen)**										
**IMSTAR-HN** **NCT03700905^76^**	3 (*n* = 276)	Resectable p16- LA-HNSCC	Anti-PD-1 Anti-CTLA4	Nivolumab (Arm I)	1 cycle (2 wks)	Surgery + PORT/CRT-cisplatin	Nivolumab (Arm Ia)	6 mos	DFS	Unknown status
							Nivolumab + ipilimumab (Arm Ib)	6 mos		
				None (Arm II)			–	–		
**NCT04080804^77^**	2 (*n* = 80)	Resectable, cStage III-IVa	Anti-PD-1 Anti-LAG3 Anti-CTLA4	Nivolumab + relatlimab	1 cycle (4 wks)	Surgery	-	-	Adverse events, ORR^c^, pRR^d^	Recruiting
				Nivolumab + ipilimumab						
**NCT06170710^78^**	2 (*n* = 173)	High risk resected HNSCC	Various Anti-PD-1	Anti-PD-1	N/A	Surgery + PORT + cisplatin	Anti-PD-1 Ab (pembrolizumab, nivolumab, tislelizumab, camrelizumab, sintilimumab, toliparimab)	N/A	2-yr DFS	Recruiting
**NCT06804850^79^**	2 (*n* = 98)	Resectable	Anti-PD-1 EGFR TKI Pre-op low-dose RT	Tislelizumab + afatinib + low dose RT 4 Gy/2Fx	2 cycles (6 wks)	Standard of care surgery	-	-	MPR^a^	Recruiting
				Tislelizumab + afatinib						
**NCT06814067^80^**	2 (*n* = 80)	Resectable, cStage III-IVB	Anti PD-1/VEGF bispecific	Pembrolizumab	2 cycles (6 wks)	Surgery + PORT ± cisplatin	Pembrolizumab	15 cycles (45 wks)	MPR^a^	Not yet recruiting
				Ivonescimab			Ivonescimab			
**Adjuvant ICI**										
**RTOG-1216** **NCT01810913^81^**	2/3 (*n* = 613)	Resected pStage III/IV with high risk (ENE or R1)	Anti-EGFR Anti-PD-L1			Surgery + PORT + cisplatin	–	–	Phase 2: DFS Phase 3: OS	Recruiting
						Surgery + PORT + cisplatin	Atezolizumab	6 cycles (18 wks)		
						Surgery + PORT + docetaxel + cetuximab	–	–		
						Surgery + PORT + docetaxel	–	–		

a pCR = pathological complete response.

b MPR = major pathologic response.

c ORR = objective response rate.

d pRR = MPR + pCR.

For unresectable LA-HNSCC, current studies focus on two main strategies (Table 4^99–105^ and Supplementary Table S3^106–113^): adding ICIs to standard CRT—either during concurrent RT, adjuvant maintenance, or both^102–107^—and radioimmunotherapy approaches combining ICIs with RT and/or maintenance.^108–110^ Large phase III trials are ongoing, including eVOLVE-HNSCC (adjuvant bispecific anti-PD-1/CTLA-4 after CRT),^102^ JADE (dostarlimab adjuvant maintenance in PD-L1-positive patients),^103^ and pembrolizumab with or without lenvatinib post-CRT in CPS ≥ 1 populations.^111^ Additional studies are exploring induction ICIs or ICI-based combinations with chemotherapy^99^^,^^112^^,^^113^ or targeted therapy (e.g., olaparib) followed by definitive CRT or radioimmunotherapy, with or without adjuvant ICIs.^100^^,^^101^

**Table 4.** Phase III and selected ongoing phase II studies of induction and/or adjuvant IO study with definitive CRT in HNSCC.

Study	Phase (*n*)	Population	Investigational objective	Induction	Duration of induction	Definitive treatment	Adjuvant	Duration of maintenance adjuvant	1EP	Recruitment status
**Induction ICI-based regimen**										
**NCT06345287^99^**	2 (*n* = 133)	LA non-HPV related, post-induction chemoimmunotherapy and not suitable for surgery	Chemotherapy Anti-PD-1/PD-L1 Response adaptive CRT	Chemotherapy and Anti-PD1/PDL1	2-4 cycles (6-12 wks)	CRT + Anti-PD1/PDL1 CR: 60 Gy deep PR: 66 Gy non-deep PR: 70 Gy	Anti-PD1/PDL1	17 cycles (1 yr)	PFS	Recruiting
**NCT05366166^100^**	2 (*n* = 45)	cStage III-IVB	Anti-PD-1 PARP inhibitor	Pembrolizumab + Olaparib	1 cycle (6 wks)	Cisplatin-RT	Pembrolizumab + Olaparib	8 cycles (48 wks)	PFS	Recruiting
**NCT07041788^101^**	2 (*n* = 25)	Unresectable bulky	SFRT Anti-PD-1 Anti-CTLA4 Chemotherapy	SFRT (10-20Gy/1Fx) + docetaxel + cisplatin + Iparomlimab + Tuvonralimab	3 cycles (9 wks)	Cisplatin-RT	Iparomlimab + Tuvonralimab	14 cycles (42 wks or 1 yr)	2-yr EFS	Not yet recruiting
**Adjuvant ICI-based regimen**										
**eVOLVE-HNSCC** **NCT06129864^102^**	3 (*n* = 1145)	LA unresectable cStage III-IVB after definitive CRT	Anti-PD-1/CTLA4 bispecific	None	-	Cisplatin-RT	Volrustomig	18 cycles (1 yr)	PFS in PD-L1+ tumor	Recruiting
							Observation			
**JADE** **NCT06256588^103^**	3 (*n* = 864)	PDL-1+ CPS ≥ 1 LA unresectable	Anti-PD-1			Cisplatin-RT	Dostalimab	15 cycles (1 yr)	EFS	Recruiting
							Observation			
**CATCH** **NCT04861467^104^**	2 (*n* = 155)	cStage III-IVA, CR/PR/SD after definitive CRT				CRT	Camrelizumab	N/A	PFS	Not yet recruiting
							Placebo			
**NCT03383094^105^**	2 (*n* = 114)	High-intermediate risk p16+ unresectable				Cisplatin-RT + Pembrolizumab	Pembrolizumab	17 cycles (1 yr)	PFS	Recruiting
						Cisplatin-RT	Observation	–		

#### Ongoing studies of ICIs for de-escalation and response-adaptive RT

In this context, response-adapted RT (Table 5^114–117^ and Supplementary Table S4^118–120^) refers to de-escalation strategies—such as omission of PORT or reductions in dose, target volume, or fractionation of definitive RT/CRT—in patients with favorable responses to ICI-based neoadjuvant therapy, with the aim of minimizing RT-related toxicity.

**Table 5.** Phase III and selected ongoing phase II studies of surgical conversion and response adaptive definitive treatment either surgery or CRT after induction treatment.

Study	Phase (*n*)	Population	Investigational treatment	Induction	Duration of induction	Definitive treatment	Adjuvant	Duration of maintenance adjuvant	1EP	Recruitment status
**PRECEDE** **NCT06957938^114^**	3 (*n* = 168)	LA-SCC of larynx/HP amenable to TLG	Anti-PD-1 Chemotherapy Organ preservation	Tislelizumab + cisplatin + nab-paclitaxel	3 cycles (9 wks)	Cisplatin-RT Surgery + PORT ± cisplatin	-	-	2-yr LFSR^b^	Not yet recruiting
				Cisplatin + nab-paclitaxel						
**NCT06100497^115^**	2 (*n* = 100)	cStage III-IVB poorly differentiated (2 cohorts)	Anti-PD-1 Chemotherapy Respond adaptive PORT	Pembrolizumab + cisplatin + nab-paclitaxel	3 cycles (9 wks)	Cohort cStage IVB: Surgery + RT ± cisplatin or definitive cisplatin-RT	Pembrolizumab	14 cycles (42 wks)	ORR^a^	Recruiting
					2-3 cycles (6-9 wks)	Cohort cStage III-IVA Surgery + RT ± cisplatin If pCR → omit PORT^c^		14-15 cycles (42-45 wks)		
**NCT05272696^116^**	2 (*n* = 86)	Resectable, cStage III-IVA	Anti-PD-1 Chemotherapy	Pembrolizumab + nab-paclitaxel + cisplatin	2 cycles (6 wks)	If PR/CR → definitive CRT If SD/PD → Surgery ± PORT	Only for patients with high-risk factors (not specified)	N/A	ORR^a^	Recruiting
**NCT06611137^117^**	2 (*n* = 35)	LA-SCC larynx/HP cStage III-IVB	Pre-op SBRT Anti-PD-1 Chemotherapy	SBRT + Toripalimab + docetaxel + cisplatin	3 cycles (9 wks)	If CR/PR → definitive IMRT (54 Gy/27Fx) If SD/PD → Surgery ± PORT	–	–	ORR^a^	Recruiting

a ORR = objective response rate.

b LFSR = laryngectomy-free survival rate

c pCR = pathological complete response

In trials such as KEYNOTE-689, all patients receiving perioperative pembrolizumab are required to undergo postoperative (chemo)radiotherapy regardless of their pathological response. While this approach has demonstrated clinical benefit, it may also result in overtreatment for a subset of patients, particularly when compared with the conventional paradigm in which postoperative RT or CRT is reserved for patients with established pathological risk factors. This provides a rationale for response-adapted postoperative strategies that may preserve oncologic efficacy while reducing treatment intensity and long-term toxicity in selected patients with favorable responses. However, evidence remains limited; small phase II studies have explored this approach, but results are inconclusive and long-term outcomes are lacking.^19–22^ Several ongoing trials are evaluating response-adapted PORT in patients achieving pCR or MPR after induction ICI-based therapy, while maintaining or intensifying treatment in those with residual or high-risk disease.^74^^,^^75^^,^^115^^,^^116^ To date, response-adaptive PORT is not currently recommended in routine practice due to insufficient evidence.

In the definitive setting, dose-adapted RT following induction chemoimmunotherapy is also under investigation.^99^ However, these approaches remain investigational, and their safety, reproducibility, and long-term oncologic outcomes require prospective validation.

#### Ongoing studies of ICIs for surgical conversion in borderline resectable LA-HNSCC and organ preservation

Conversion therapy for borderline resectable or unresectable HNSCC remains challenging (Table 5). Two phase II studies are evaluating induction ICIs combined with chemotherapy followed by definitive treatment based on post-induction resectability.^115^^,^^118^ In laryngeal and hypopharyngeal cancers, organ preservation strategies must balance survival outcomes with function preservation and typically involve definitive CRT or induction chemotherapy followed by RT in responders.^121^ Ongoing trials are investigating whether induction chemoimmunotherapy could improve upon induction chemotherapy alone with subsequent response-guided definitive treatments and continued adjuvant ICIs per study protocol.^114^^,^^117^^,^^119^

## Role of (neo)adjuvant therapeutic cancer vaccines

Several early-phase studies have explored therapeutic vaccines in non-metastatic HNSCC. A phase I/II study investigated a viral-based personalized neoantigen vaccine (TG4050) in the adjuvant setting after surgery and PORT/CRT in HPV-negative LA-HNSCC (pathological stages III-IV).^122^ Oral cavity primaries accounted for 73%, and high-risk features (ECE or positive margins) were present in 57%. Patients were randomized to early vaccine initiation or delayed initiation at recurrence. TG4050 was given as six weekly injections followed by 14 injections every 3 weeks. No dose-limiting toxicities occurred, and durable CD8+ T-cell responses persisted >1 year, with 2-year DFS of 100% and 81%, respectively. The phase II portion is ongoing. Another phase II study evaluated a neoadjuvant HPV DNA vaccine with pembrolizumab and interleukin-7 in resectable HPV16/18-positive HNSCC, followed by surgery and PORT/CRT.^123^ Among 11 patients, MPR and pCR rates were 63.6% and 36.3%, respectively. A phase I study assessed adjuvant autologous dendritic cell vaccines targeting p53 in HLA-A2.1-positive resectable HNSCC and reported encouraging DFS despite modest p53-specific immune responses.^124^

Most ongoing therapeutic vaccine trials focus on HPV-positive HNSCC, primarily targeting the viral oncoproteins E6 and E7 as tumor-specific antigens. Vaccines are being investigated in both neoadjuvant settings—alone^125–127^ or in combination with chemotherapy^128^^,^^129^ or ICIs^130^^,^^131^—and in the adjuvant setting.^132^^,^^133^ The combination of therapeutic vaccination with concurrent cisplatin-CRT and pembrolizumab is also under active investigation.^134^

## Biomarkers and precise patient selection

Beyond PD-L1 status, biomarker-driven treatment strategies, including the use of circulating-tumor DNA (ctDNA)-based minimal residual disease (MRD) assessment, represent one of the most promising future directions in the field. The concept of tailoring immunotherapy in the locally advanced setting according to MRD status is particularly attractive, as it may help identify patients who are most likely to benefit from treatment intensification while potentially sparing others from unnecessary therapy.

Current approaches include circulating tumor HPV-DNA (ctHPV-DNA) assays for HPV-related HNSCC and tumor-informed or tumor-agnostic ctDNA assays for HPV-unrelated disease. Multiple studies support the feasibility and prognostic value of ctDNA-based MRD detection, although most are limited by small sample sizes and heterogeneous, non-standardized methodologies. Persistent or reemergent ctDNA after treatment is consistently associated with inferior PFS and OS and may precede clinically detectable recurrence by weeks to months.^135–138^ In HPV-positive HNSCC, ctHPV-DNA demonstrates high sensitivity and specificity for recurrence detection and is closer to clinical implementation. In contrast, ctDNA assays in HPV-negative disease remain more challenging. In the LIONESS study using a tumor-informed approach, 91% of patients with serially detectable ctDNA more than 2 weeks after surgery developed recurrence, with a lead time of up to 500 days; however, assay sensitivity may be limited by biological and intratumoral heterogeneity.^139^

Ongoing studies in LA-HNSCC are largely early-phase or observational and focus on prognostic utility, recurrence prediction, and the feasibility of ctDNA-guided strategies, including RT de-escalation—particularly in HPV-related OPSCC—and selective use of adjuvant therapy.^140–145^ ctDNA is also being incorporated into response-adaptive frameworks; for example, a phase II trial administers adjuvant ICIs selectively to patients with detectable ctDNA following induction therapy.^72^

Despite these advances, ctDNA currently functions primarily as a prognostic biomarker, and its ability to predict benefit from ICI-based therapy or guide MRD-directed escalation or de-escalation remains to be established. Larger prospective studies are needed to define clinically relevant thresholds, standardize assays and sampling strategies, and address variability in tumor shedding and heterogeneity across disease subsites. Integration with additional biomarkers—such as tumor mutational burden, gene-expression signatures, and spatial immune profiling—may further enhance risk stratification and refine patient selection in future ICI trials.^146–149^

## Conclusion

The integration of ICIs into the management of resectable HNSCC represents a paradigm shift, as demonstrated by the KEYNOTE-689 and NIVOPOSTOP studies. However, optimal sequencing, patient selection, and treatment duration remain under investigation. Moreover, the role of ICIs in the R/M setting among patients who experience disease progression after (neo)adjuvant ICIs for resectable LA disease remains unclear and is a critical area of ongoing research. Response-adaptive strategies, organ-preservation approaches, treatment de-escalation, and surgical conversion—along with combination regimens involving chemotherapy, antibody–drug conjugates, targeted therapy, radiotherapy, or novel immunotherapies—are being explored to enhance efficacy while minimizing toxicity and improving quality of life. Therapeutic cancer vaccines, particularly in HPV-related HNSCC or neoantigen-targeted settings, hold promise as adjunctive neoadjuvant or adjuvant treatments, although outcomes from early-phase clinical trials remain preliminary. Key challenges persist, including the management of postoperative low-risk patients, defining ICI-refractory disease, and integrating ICIs into unresectable HNSCC treated with definitive RT/CRT. Future studies are expected to refine precise patient selection, optimize combination strategies, and establish more precise, individualized treatment paradigms for HNSCC.

## Supplementary Material

oyag366_Supplementary_Data

## Data Availability

No new data were generated or analyzed in support of this review. All information reviewed in this article is available in the cited references.
