# Supplementary material for: Immune checkpoint inhibitors in resectable and locoregionally advanced HNSCC: past lessons, present evidence, and future directions
Source: Oncologist. 2026 Sep 17;31(11):oyag366. doi: 10.1093/oncolo/oyag366 (PMC13645433; doi:10.1093/oncolo/oyag366)
Supplement: Supplementary file 1 [file oyag366_supplementary_data.docx]

**Supplement**

**Table S1**. Comparison of potential practice changing IO studies in resectable HNSCC patients (Perioperative KN689 vs Adjuvant NIVOPOSTOP study)

| **Study** | **KN689** ^[16]^ | | **NIVOPOSTOP** ^[17]^ | |
| --- | --- | --- | --- | --- |
| **Arms** | **Pembrolizumab  (n=363)** | **SOC  (n=351)** | **Nivolumab  (n=332)** | **SOC (n=334)** |
| Population | Resectable cStage III-IVA HNSCC | | Resected pStage III-IVB with high-risk features HNSCC | |
| Definitive treatment | Surgery | | Surgery | |
| Primary (Oral/HP/Larynx/OP) | 60/22/8/10 | 61/21/7/11 | 58/13/12/17 | 58/12/12/18 |
| HPV positive | 3% | 4% | 5% | 5% |
| PD-L1 status | CPS < 1 = 4% CPS ≥ 1 = 96% CPS ≥ 10 = 65% | | CPS < 1 = 12% CPS 1-19 = 44% CPS ≥ 20 = 37% | |
| Arms | - Induction pembrolizumab - PORT ± cisplatin + pembrolizumab - Adjuvant pembrolizumab | PORT ± cisplatin | - PORT + cisplatin + nivolumab - Adjuvant nivolumab | PORT + cisplatin |
| Duration of IO | 17 cycles (1 yr) | - | 10 cycles (30 wks) | - |
| High risk features |  | |  | |
| - ECE or R1 | 32.5% | 44.4% | 57% | 59% |
| - ECE and R1 | N/A | | 33% | 30% |
| - Multiple PNI or ≥4 LN+ |  |  | 10% | 11% |
| Median EFS^a^ /DFS^a^ (HR [95%CI]; p-value) | 51.8 vs 30.4 (mos)  HR 0.73 (0.58 – 0.92); p 0.008 | | NR  HR 0.76 (0.60 – 0.98); p 0.034 | |
| Median Follow-up (mo) | 38.3 | | 30.3 | |
| Locoregional recurrence | 11% | 11% | 13%^c^ | 20%^c^ |
| Distant recurrence | 7% | 15% | 13%^c^ | 13%^c^ |
| OS  (HR [95%CI]; p-value) | 3-yr: 68.4% vs 61.1%  0.72 (0.52 – 0.98); p 0.04^b^ (CPS-10) | | 3-yr: 74.2% vs 67.8% | |

^a^primary endpoint, ^b^not significant at IA1, ^c^cumulative incidence at 3 years

**Table S2**. Additional ongoing phase II studies of induction and/or adjuvant ICIs with definitive surgery

| **Study** | **Phase**  **(n)** | **Population** | **Investigational Treatment** | **Induction** | **Duration of Induction** | **Definitive treatment** | **Adjuvant Immunotherapy** | **Duration of Maintenance Adjuvant** | **1EP** | **Recruitment status** |
| --- | --- | --- | --- | --- | --- | --- | --- | --- | --- | --- |
| **Induction ICI with chemotherapy-based regimen** | | | | | | | | | | |
| **HNIC21**  NCT04922450  ^[80]^ | 2  (n=53) | Resectable LA-HNSCC | - Anti-PD-1 | Camrelizumab + albumin paclitaxel + cisplatin | 3 cycles  (9 wks) | Surgery + PORT with chemotherapy | - | - | CRR^e^, PRR^f^ | Unknown status |
| **Neo-TIME**  NCT05862168  ^[84]^ | 2  (n=30) | Resectable cStage III-IVb OCSCC |  | Tislelizumab + nab-paclitaxel + cisplatin + 5FU | 3 cycles  (9 wks) | Surgery | - | - | pCR^c^ | Recruiting |
| NCT06235918  ^[85]^ | 2  (n=30) | Resectable HNSCC |  | Tislelizumab + nab-paclitaxel + carboplatin | 2 cycles  (6 wks) | Surgery | - | - | pCR^c^ | Recruiting |
| **NeoSPOT**  NCT06009861  ^[83]^ | 2  (n=110) | Resectable LA cStage III-IV OCSCC/OPSCC |  | Tislelizumab + nab-paclitaxel + cisplatin | 2 cycles  (6 wks) | Surgery + PORT/CRT | Tislelizumab in high risk | 14 cycles  (42 wks) | EFS | Active, not recruiting |
| **NeoTempo**  NCT07147426  ^[94]^ | 2  (n=100) | Resectable cStage III-IVB HNSCC |  | Tislelizumab + nab-paclitaxel + cisplatin | 4 cycles (12 wks) | Surgery | - | - | pCR^c^ | Not yet recruiting |
|  |  |  |  |  | 2 cycles  (6 wks) |  |  |  |  |  |
| NCT05446467  ^[81]^ | 2  (n=80) | Resectable cStage III-IVA LA-HNSCC |  | Pembrolizumab + 5FU + low-dose cisplatin | 6 cycles  (18 wks) | Surgery + PORT/CRT | Pembrolizumab | N/A | pCR^c^ of primary site | Recruiting |
| NCT06277791  ^[82]^ | 2  (n=35) | cStage IVB oral SCC | - Anti-PD-L1 | Adebrelimab+ docetaxel + cisplatin | 3 cycles  (9 wks) | Surgery + PORT/CRT | Adebrelimab, based on patients’ condition | N/A | pCR^c^+MPR^a^ | Recruiting |
| **Induction ICI and targeted therapy and ICI combination (non-chemotherapy regimen)** | | | | | | | | | | |
| NCT05993858  ^[86]^ | 2  (n=33) | Resectable cStage III-IVb HNSCC | - Anti-PD-1 - Anti-EGFR | Toripalimab + cetuximab | 3 cycles  (9 wks) | Surgery + PORT + cisplatin | Toripalimab | 17 cycles  (51 wks) | pCR^c^ | Not yet recruiting |
| **Neo-AdDa**  NCT06199271  ^[88]^ | 2  (n=30) | Resectable LA-HNSCC cStage III-IVB | - Anti-PD-L1 - CDK 4/6 inhibitors | Adebrelimab +  dalpiciclib | 3 cycles  (9 wks) | Surgery | - | - | pRR^b^ | Not yet recruiting |
| NCT03708224  ^[91]^ | 2  (n=55) | Resectable HNSCC | - Anti-PD-L1 - Anti-TIGIT - Anti-IL-6 - Benefit of adjuvant Anti-PD-L1 | Atezolizumab | 2 cycles  (6 wks) | Surgery + PORT/CRT | - | - | Patients with increased differentiation CD3, R0 resection | Recruiting |
|  |  |  |  | Atezolizumab |  |  | Atezolizumab | 12 cycles  (36 wks) |  |  |
|  |  |  |  | Atezolizumab + Tiragolumab |  |  | - | - |  |  |
|  |  |  |  | Atezolizumab + Tocilizumab |  |  |  |  |  |  |
| **Icemelting-2**  NCT05069857  ^[87]^ | 2  (n=46) | Resectable cStage III-IVA OCSCC with PD-L1 CPS > 10 | - Anti-PD-1 - anti-VEGFR | Camrelizumab  + apatinib | 3 cycles  (6 wks) | Surgery + PORT/CRT | - | - | DFS | Recruiting |
| **NEOSTART**  NCT06673693  ^[90]^ | 2  (n=24) | Resectable cStage III-IVB HPV negative HNSCC | - Anti-PD-1 - Pre-Op SBRT | Tislelizumab + SBRT | 2 cycles  (6 wks) | Surgery | - | - | MPR^a^ | Recruiting |
| **2023HNRT01**  NCT05861557  ^[89]^ | 2  (n=23) | Resectable LA-HNSCC |  | Toripalimab + SBRT | 2 cycles  (6 wks) | Surgery + PORT/CRT | Toripalimab | 8 cycles  (6 mos) | MPR^a^ | Unknown status |
| **NICO**  NCT03721757  ^[95]^ | 2  (n=21) | Resectable OCSCC | - Anti-PD-1 | Nivolumab | 1 cycle  (2 wks) | Surgery + PORT/CRT | Nivolumab | 6 cycles (24 wks) | DFS | Completed |
| **Adjuvant ICI** | | | | | | | | | | |
| NCT04992559  ^[92]^ | 2  (n=41) | Post-op high risk LA HNSCC after adjuvant (C)RT | - Anti-PD-1 | - | - | Surgery + PORT/CRT | Toripalimab | 16 cycles  (12 mos) | 2-yr DFS | Recruiting |
| **NFEC-2022-371**  NCT05773079  ^[93]^ | 2  (n=28) | High risk LA-HNSCC definitive treatment without residual lesions |  |  |  | SOC (Surgery +/- PORT/CRT, and including definitive RT/CRT) | Toripalimab | 17 cycles  (1 yr) | 2-yr PFS | Not yet recruiting |
| NCT03529422  ^[96]^ | 2  (n=18) | Resected intermediate risk HNSCC | - Anti-PD-L1 |  |  | Surgery | Durvalumab-RT | 3 cycles  (9 wks) | 3-yr DFS | Active, not recruiting |

^a^MPR = Major Pathologic Response; ^b^pRR = MPR+pCR; ^c^pCR = pathological complete response; ^d^ORR = objective response rate; ^e^CRR = Clinical remission rate; ^f^PRR = Pathologic remission rate.

**Table S3.** Additional ongoing phase II studies of induction and/or adjuvant IO study with definitive CRT

| **Study** | **Phase**  **(n)** | **Population** | **Investigational Objective** | **Induction** | **Duration of Induction** | **Definitive treatment** | **Adjuvant** | **Duration of Maintenance Adjuvant** | **1EP** | **Recruitment status** |
| --- | --- | --- | --- | --- | --- | --- | --- | --- | --- | --- |
| **Induction ICI-based regimen** | | | | | | | | | | |
| **SMART-KEY**  LACOG 0720  NCT04943445  ^[111]^ | 2  (n=43) | cStage III-IVB laryngeal SCC (glottic/supreglottic) | - Anti-PD1 - Chemotherapy | Pembrolizumab + carboplatin + paclitaxel | 3 cycles  (9 wks) | Pembrolizumab-RT | Pembrolizumab | 12 cycles  (36 wks) | 2-yrs LFS | Active, not recruiting |
| **CRIA**  NCT05213884  ^[110]^ | 2  (n= 30) | Unresectable cStage III-IVB LA-HNSCC | - Anti-PD1 | Camrelizumab | 1-3 cycles  (3-9 wks) | Cisplatin-CRT | Camrelizumab | 16 cycles  (1 yr) | PFS | Recruiting |
| **Adjuvant ICI-based regimen** | | | | | | | | | | |
| **REWRITe**  NCT03726775  ^[106]^ | 2  (n=61) | N0 early and LA-HNSCC | - Anti-PDL1 - Omission of large prophylactic neck irradiation | None | - | Durvalumab-RT | Durvalumab | 6 cycles  (6 mos) | Regional (neck) nodal control rate | Active, not recruiting |
| NCT05433116  ^[109]^ | 2  (n=50) | LA-HNSCC cStage III-IVB PDL-1 CPS ≥1 after definitive CRT | - Anti-PD1 - Multi-kinase VEGF TKI |  |  | CRT | Pembrolizumab + Lenvatinib | 17 cycles  (51 wks) | 2-yr EFS | Recruiting |
|  |  |  |  |  |  |  | Pembrolizumab |  |  |  |
| NCT03532737  ^[105]^ | 2  (n=50) | LA-HNSCC cStage III-IVA | - Anti-PD1 |  |  | Cisplatin-CRT or Cetuximab-RT + Pembrolizumab | Pembrolizumab | 6 cycles  (18 wks) | Dose-limiting toxicity and ORR^d^ | Active, not recruiting |
| NCT04405154  ^[104]^ | 2  (n=32) | LA unresectable HNSCC |  |  |  | Cisplatin-CRT + Camrelizumab | - | 3 cycles  (6 wks) | ORR^d^ | Unknown status |
| **DURTRE-RAD**  NCT03624231  ^[107]^ | 2  (n=18) | LA unresectable HNSCC | - Anti-PDL1 - Anti CTLA4 |  |  | Durvalumab-RT | Durvalumab | 10 cycles  (40 wks) | PFS | Completed |
|  |  |  |  |  |  | Durvalumab/Tremelimumab-RT | Durvalumab/Tremelimumab  (Tremelimumab up to 4 cycles) |  |  |  |
| **CITHARE**  NCT03623646  ^[108]^ | 2  (n=11) | Early-stage HPV+ OPSCC eligible for definitive CRT | - Anti-PD-L1 |  |  | Cisplatin-CRT | Observation | - | 12-mo PFS | Completed |
|  |  |  |  |  |  | Durvalumab-RT | Durvalumab | N/A |  |  |

^d^ORR = objective response rate

**Table S4.** Additional ongoing phase II studies of surgical conversion and response adaptive definitive treatments (surgery or CRT) after induction treatment

| **Study** | **Phase**  **(n)** | **Population** | **Investigational Treatment** | **Induction** | **Duration of Induction** | **Definitive treatment** | **Adjuvant** | **Duration of Maintenance Adjuvant** | **1EP** | **Recruitment status** |
| --- | --- | --- | --- | --- | --- | --- | --- | --- | --- | --- |
| NCT06978829  ^[118]^ | 2  (n=42) | LA unresectable HNSCC | - Anti-PD-1 - Chemotherapy - Anti-EGFR | Tislelizumab + platinum + nab-paclitaxel + cetuximab | 3 cycles  (9 wks) | If operable → surgery + adjuvant chemotherapy  If inoperable → definitive RT | Tislelizumab | 17 cycles  (1 yr) | ORR^d^ | Recruiting |
| **TREC2023-KY073**  NCT06725498  ^[116]^ | 2  (n=15) | Unresectable cT4b HNSCC | - Anti-PD-1 - Intraarterial chemotherapy (IAC) | Tislelizumab + cisplatin + albumin-bound paclitaxel | 3 cycles  (9 wks) | If operable → Surgery ± PORT/CRT  If inoperable → definitive RT with concurrent IAC | Tislelizumab | 17 cycles  (1 yr) | 1-yr EFS | Recruiting |
| NCT03894891  ^[117]^ | 2  (n=6) | LA resectable or unresectable larynx/HP SCC cStage III-IV | - Anti-PD-1 - Chemotherapy | Nivolumab + docetaxel + cisplatin | 3 cycles  (9 wks) | If PR/CR → Nivolumab-RT  If SD/PD → Salvage laryngectomy and/or pharyngectomy | Nivolumab | 6 cycles  (24 wks) | LFS | Completed |

^d^ORR = objective response rate
